# Optimal Configuration for Monitoring Stations in a Wireless Localisation Network Based on Received Signal Strength Differences

**DOI:** 10.3390/s23031150

**Published:** 2023-01-19

**Authors:** Mehdi Eshagh

**Affiliations:** Department of Engineering Science, University West, 461 86 Trollhättan, Sweden; mehdi.eshagh@hv.se

**Keywords:** jamming, least-squares method, quadratic optimisation, spoofing, variance-covariance matrix

## Abstract

A smart city is a city equipped with many sensors communicating with each other for different purposes. Cybersecurity and signal security are important in such cities, especially for airports and harbours. Any signal interference or attack on the navigation of autonomous vehicles and aircraft may lead to catastrophes and risks in people’s lives. Therefore, it is of tremendous importance to develop wireless security networks for the localisation of any radio frequency interferer in smart cities. Time of arrival, angle of arrival, time-difference of arrivals, received signal strength and received signal strength difference (RSSD) are known observables used for the localisation of a signal interferer. Localisation means to estimate the coordinates of an interferer from some established monitoring stations and sensors receiving such measurements from an interferer. The main goal of this study is to optimise the geometric configuration of the monitoring stations using a desired dilution of precision and/or variance-covariance matrix (VCM) for the transmitter’s location based on the RSSD. The required mathematical models are developed and applied to the Arlanda international airport of Sweden. Our numerical tests show that the same configuration is achieved based on dilution of precision and VCM criteria when the resolution of design is lower than 20 m in the presence of the same constraints. The choice of the pathloss exponent in the mathematical models of the RSSDs is not important for such low resolutions. Finally, optimisation based on the VCM is recommended because of its larger redundancy and flexibility in selecting different desired variances and covariances for the coordinates of the transmitter.

## 1. Introduction

These days the world is experiencing global warming, and one of the ways of tackling this issue is to create smart cities and autonomous vehicles. A smart city is a city equipped with different types of active and passive sensors communicating with each other. Cyber and signal security are tremendously important to keep a smart city safe and prevent any cyber and signal attacks. This is even more important for autonomous vehicles and their navigation. Therefore, a smart city should be equipped well with security networks for the localisation of any signal interference device. In this article, the design of such a security network based on the received signal strength difference (RSSD) is presented.

Two known types of signal interference are jamming and spoofing. The former means to transmit a signal into the same or near band as the satellite navigation band to disable navigation and spoofing stands for transmitting a fake signal [1]. Studies have shown that a simple and cheap spoofer can overtake for e.g., a ship navigation without being detected; see [2,3]. Since the power level of the global navigation satellite systems (GNSS) signal is low, therefore, a weak interference signal can jam a receiver [1], see some real examples in [4,5,6,7,8,9,10,11].

Localisation of an interferer means estimating its coordinates in a pre-defined coordinate system. Some points with known coordinates, which are so-called anchor nodes or monitoring stations (MSs) have some sensors to receive information from the interferer. If the sensors can detect an interfering signal and measure its time of arrival (TOA), angle of arrival (AOA) or time difference of arrivals (TDOA) the interfering device can be localised. Numerous studies have been conducted for localisation based on these observables, e.g., see [12,13,14].

Another type of information, which can be received from the interferer is known as pathloss information such as received signal strength (RSS), RSSD, or received signal voltage. Thompson et al. [14] tested an outdoor localisation of a Wifi with unknown transmission power based on the RSS and concluded that in their 140 m^2^ test area the source can be localised with a root mean squares error of about 37 m. Thompson et al. [15] used automatic gain control [16] for the detection and estimation of jammer to noise of an interference and concluded that their method results in the capability of detecting jammer-to-noise ratios down to −8 dB. Wang and Inkol [17] developed an efficient algorithm for solving the nonlinear problem of localisation using the RSSD. Thompson et al. [18] developed the localisation procedure based on the RSS in the presence of ground reflection. Thompson [19] investigated the interference device detection and localisation by analysing the dilution of precision (DOP), from the RSS, TDOA and concluded that the TDOA is superior to the RSS. Bekcibasi and Tenruh [20] presented a method for increasing the accuracy of localisation based on the RSS and named it dynamic distance reference anchor method. Egenbråten [21] studied radio frequency emitter localisation based on the power difference of arrivals. Robertson et al. [22] performed a similar study on a Network of GNU Radio Sensors. Hu and Leus [23] presented a robust localisation method based on the RSSD and the semidefinite programming. Nyström [24] also studied the localisation of the GNSS interference using the RSSD. Zhou et al. [25] studied the error propagation for both mobile and target tracking and sensor node location calibration. Localisation using the RSS was also studied by Niu et al. [26] with focus on practical issues and solutions. Pu and You [27] proposed a location fingerprinting algorithm based on the general and weighted k-nearest neighbour algorithms to estimate the position of the target node using the RSS with an estimated position error of about 1.8 m. Li et al. [28] used the orientation compensation method and the RSSD for localisation and mentioned that their approach was effective in enhancing localisation that had multiple devices with three-dimensional orientation diversity. Wu et al. [29] presented a positioning approach based on linear regression of the AOA and the RSSD and showed that the proposed algorithm can achieve better accuracy than existing Radio Frequency Identification (RFID) positioning approaches. Xu and Dogancay [30] suggested a method for estimating the optimal geometric configuration of sensors for the three-dimensional localisation of targets using RSS measurements. The proposed evaluation function has been solved using successive optimisation algorithms to obtain optimal positions of the sensors. Xu [31] investigated the optimal sensor placement strategy for single static target localisation using the hybrid RSS, AOA and TOA measurements on the two-dimensional plane. Lee et al. [32] proposed a hybrid localisation algorithm to boost the accuracy of range-based localisation; they replaced the ranging part of the rule-based localisation method with a deep regression model that uses data-driven learning with the dual-band RSS. Bo et al. [33] studied the configuration of sensors for the cooperative localisation of autonomous underwater vehicles using the RSS. Alanezi et al. [34] proposed a method to accurately determine the position and distance of the wireless sensors linked in a local network. The method utilises the RSS at the target node to identify its location in the localised grid system.

The location of the MSs or in other words, their geometric configuration has a significant role in the quality of localisation. Xu [31] studied this issue for the hybrid RSS, AOA, and TOA, but Eshagh [35] applied quadratic optimisation with constraint for obtaining an optimal configuration for the MSs based on the AOA and the TDOA, and concluded the optimal design based on the TDOA is highly dependent on the geometric form of the area, and his efforts for design optimal configuration for the Landvetter airport of Sweden was not successful. However, in another study Eshagh [36] showed that optimisation can be performed without problem if the area is close to a square and applied to the Arlanda International airport in Sweden.

This study aims to develop a method, provide all mathematical tools, and show how to apply them for the optimal design of a wireless localisation security network based on the RSSDs from a signal interference device. To do so, a configuration of a four-MS localisation network is considered and optimised using the predefined variance-covariance matrix (VCM) or the dilution of precision (DOP) for the coordinates of all nodes of a grid covering a study area. In addition, this paper shows that simply by selecting a suitable place for the MSs, the quality of localisation can significantly be improved without any extra cost. In Section 2, the principle of the RSSD is presented, and after that, in Section 3 the problem of localisation using the RSSD is provided. Section 4 deals with mathematical developments of the optimisation criteria VCM and DOP for a localisation network based on the RSSD. Section 5 discusses the optimisation model and the required limiting constraints. In Section 6, the presented design method is applied to the Arlanda international airport in Sweden.

## 2. Received Signal Strength Difference

RSS depends on how long the signal has been on its way to the receiver, in other words, the distance from the transmitter. This RSS ($P_{r}$) has the following mathematical formula [17]:

(1) $$P_{r}=\frac{A\left(h_{t},h_{r},f\right)}{d^{\gamma }}P_{t}$$

where $P_{t}$ is the signal strength at the transmitter, $A\left(h_{t},h_{r},f\right)$ is a parameter, which depends on the transmitter and receiver antenna’s heights $h_{t}$ and $h_{r}$ and frequency of the radio signal *f*. γ is called pathloss exponent and *d* is the distance between the transmitter and receiver. In a two-dimensional network, the following well-known relation between the Cartesian coordinates of the transmitter and receiver exists:

(2) $$d={\left[{\left(x-x_{i}\right)}^{2}+{\left(y-y_{i}\right)}^{2}\right]}^{\frac{1}{2}}$$

where (x,y) are the coordinates of the transmitter and $\left(x_{i},y_{i}\right)$ those of the *i*^th^ MS with the receiver. By taking logarithm from both sides of Equation (1), the known equation of the RSS is derived e.g., [17]:

(3) $$\Omega =10\log_{10}\left(P_{r}\right)=10\log_{10}\left(A\left(h_{t},h_{r},f\right)P_{t}\right)-10\gamma \log_{10}\left(d\right)=C-10\gamma \log_{10}\left(d\right)$$

The first term on the right-hand side of Equation (3) can be a constant, because $P_{t}$, *f*, and $h_{t}$ are constant and if $h_{r}$ is specified in such a way that all antennas have the same height.

There are two points regarding the localisation process using Equation (3). First, *C* is unknown, and second, γ is also unknown and dependent on the environment. In practice, our goal is to estimate the coordinates of an interfering transmitter, which sends no information about its signal power and antenna’s height. In a two-dimensional localisation problem, at least, two equations are required for estimating the *x*- and *y*-coordinate of the transmitter if the parameters *C* and *γ* are known; otherwise, four equations, or MSs, are needed to simultaneously estimate the coordinates with *C* and *γ*.

An alternative way is to perform the localisation process in two steps. The first, known as calibration, is to estimate *C* and *γ* with from a priori RSS measurements over the study area. In the second step, the estimated *C* and *γ* are used and considered as constants for the RSS mathematical model (3) and the localisation process is conducted.

By writing a differential from of Equation (3), the mathematical model of the RSSD is obtained, which is the difference between the RSSs at the MSs *i* and *j* (see e.g., [20] or [17]):

(4) $$\Omega_{ij}=10\gamma \log_{10}\left(\frac{d_{i}}{d_{j}}\right)=5\gamma \log_{10}\left(\frac{{\left(x-x_{i}\right)}^{2}+{\left(y-y_{i}\right)}^{2}}{{\left(x-x_{j}\right)}^{2}+{\left(y-y_{j}\right)}^{2}}\right)$$

As Equation (4) shows, the RSSD is a function of coordinates of the MSs *i* and *j* and the transmitter in addition to γ. Here, γ can be also estimated with the coordinates of the transmitter if at least three RSSDs are measured from three MSs. Some closed-form solutions for localisation using the RSSD with three and four MSs are available in [37]. Generally, γ ranges between 1 and 6 [20]; γ = 2 is used for a free space, and γ = 4 for a flat environment. It varies between 4 and 6 for indoor environments and in some cases, such as tunnels, it is less than 2.

## 3. Localisation Based on Received Signal Strength Differences

By assuming that γ is a known constant, six observation equations of Equation (4) type can be constructed for four MSs. These mathematical models need to be linearised and solved iteratively. Their matrix form is represented by the following Gauss-Markov model (cf. [38]):

(5) $$Ax=L-\varepsilon ,\;E\left\{\varepsilon \right\}=0E\left\{\varepsilon \varepsilon^{T}\right\}=C_{L}=\sigma_{0}^{2}Q$$

where **x** is the vector of the coordinate updates to the approximate coordinates of the transmitter, and ε the vector of random errors with E{ε}=0, where E{} stands for the statistical expectation, $C_{L}$ the VCM of the observations, and finally $\sigma_{0}^{2}$ a priori variance of unit weight. The matrix **A** contains partial derivatives of the observables, the Formula (4), with respect to the unknown *x-* and *y*-coordinates of the transmitter. The general formulae for these derivatives are:

(6) $$\frac{\partial \Omega_{ij}}{\partial x}=\frac{10\gamma }{\ln\left(10\right)}\left(\frac{x-x_{i}}{d_{i}^{2}}-\frac{x-x_{j}}{d_{j}^{2}}\right)\;\mathrm{and}\;\frac{\partial \Omega_{ij}}{\partial y}=\frac{10\gamma }{\ln\left(10\right)}\left(\frac{y-y_{i}}{d_{i}^{2}}-\frac{y-y_{j}}{d_{j}^{2}}\right)$$

For the four MSs M, N, O and P, and the two unknown coordinates of the transmitter, **A** has six rows and two columns. According to the formulae of the partial derivatives presented in Equation (6), the structure of **A** is

(7) $$A=\frac{10\gamma }{\ln\left(10\right)}\left[\begin{array}{cc} \frac{x-x_{M}}{d_{M}^{2}}-\frac{x-x_{N}}{d_{N}^{2}} & \frac{y-y_{M}}{d_{M}^{2}}-\frac{y-y_{N}}{d_{N}^{2}}\\ \frac{x-x_{M}}{d_{M}^{2}}-\frac{x-x_{O}}{d_{O}^{2}} & \frac{y-x_{M}}{d_{M}^{2}}-\frac{y-y_{O}}{d_{O}^{2}}\\ \frac{x-x_{M}}{d_{M}^{2}}-\frac{x-x_{P}}{d_{P}^{2}} & \frac{y-y_{M}}{d_{M}^{2}}-\frac{y-y_{P}}{d_{P}^{2}}\\ \frac{x-x_{N}}{d_{N}^{2}}-\frac{x-x_{O}}{d_{O}^{2}} & \frac{y-y_{N}}{d_{N}^{2}}-\frac{y-y_{O}}{d_{O}^{2}}\\ \frac{x-x_{N}}{d_{N}^{2}}-\frac{x-x_{P}}{d_{P}^{2}} & \frac{y-y_{N}}{d_{N}^{2}}-\frac{y-y_{P}}{d_{P}^{2}}\\ \frac{x-x_{O}}{d_{O}^{2}}-\frac{x-x_{P}}{d_{P}^{2}} & \frac{y-x_{O}}{d_{O}^{2}}-\frac{y-y_{P}}{d_{P}^{2}} \end{array}\right]$$

In Equation (5), **L** is the vector of differences between actual and approximate observations computed from the coordinates of MSs and the approximate coordinates of the transmitter

(8) $$L=\left[\begin{array}{l} \Omega_{MN}^{}-\Omega_{MN}^{0}\\ \Omega_{MO}^{}-\Omega_{MO}^{0}\\ \Omega_{MP}^{}-\Omega_{MP}^{0}\\ \Omega_{NO}^{}-\Omega_{NO}^{0}\\ \Omega_{NP}^{}-\Omega_{NP}^{0}\\ \Omega_{OP}^{}-\Omega_{OP}^{0} \end{array}\right]=5\gamma \log_{10}\left[\begin{array}{l} d_{M}^{2}/d_{N}^{2}\\ d_{M}^{2}/d_{O}^{2}\\ d_{M}^{2}/d_{P}^{2}\\ d_{N}^{2}/d_{O}^{2}\\ d_{N}^{2}/d_{P}^{2}\\ d_{O}^{2}/d_{P}^{2} \end{array}\right]-5\gamma \log_{10}\left[\begin{array}{l} {\left(d_{M}^{0}\right)}_{}^{2}/{\left(d_{N}^{0}\right)}_{}^{2}\\ {\left(d_{M}^{0}\right)}_{}^{2}/{\left(d_{O}^{0}\right)}_{}^{2}\\ {\left(d_{M}^{0}\right)}_{}^{2}/{\left(d_{P}^{0}\right)}_{}^{2}\\ {\left(d_{N}^{0}\right)}_{}^{2}/{\left(d_{O}^{0}\right)}_{}^{2}\\ {\left(d_{N}^{0}\right)}_{}^{2}/{\left(d_{P}^{0}\right)}_{}^{2}\\ {\left(d_{O}^{0}\right)}_{}^{2}/{\left(d_{P}^{0}\right)}_{}^{2} \end{array}\right]$$

$\Omega_{ij}^{0}$, *i*.*j* = M, N, O, P are the RSSDs generated from the coordinates of the MSs and the approximate coordinates of the transmitter. $d_{i}^{0}$, *i* = M, N, O and P are the computed approximate distances from the transmitter.

The least-squares solution of Equation (5) for the vector of coordinate updates is (see e.g., [39]):

(9) $$\hat{x}={\left(A^{T}A\right)}^{-1}A^{T}L$$

where ()^T^ stands for the transposition operator of matrix algebra.

$\hat{x}$ is added to the approximate coordinates of the transmitter, and after that new **A** and **L** are computed leading to another new $\hat{x}$ is estimated. This process is iterated until the coordinate updates $\hat{x}$ do not significantly change the transmitter coordinates; in other words, the solution converges. Note that the choice of $\sigma_{0}^{2}$ has no effect on $\hat{x}$. The matrix **A** of the last iteration is used for the estimation of the VCM of the coordinates. By assuming $\sigma_{0}^{2}={\left(0.01\right)}^{2}$ and **Q** = **I**, this VCM is:

(10) $$C_{\hat{x}}={\left(0.01\right)}^{2}{\left(A^{T}A\right)}^{-1}$$

$\sigma_{0}^{2}$ can also be estimated via

(11) $${\hat{\sigma }}_{0}^{2}=\frac{{\left(L-A\hat{x}\right)}^{T}\left(L-A\hat{x}\right)}{4}$$

where the denominator 4 is the degree of freedom of the localisation problem since there are six observables and two unknowns in the created system of equations. However, the main goal of this study is to optimally design a localisation network and not localisation of a transmitter, therefore, no observations exist in our system, and $\sigma_{0}^{2}={\left(0.01\right)}^{2}$ can be simply used in our computations, because it does not affect the geometry of the localisation network.

## 4. Optimisation Criteria for Localisation Networks

For designing or analysing a localisation network a grid of points covering the study area is designed, where each node of the grid is a representative of the probable location of the interfering transmitter. A local coordinate system is simply defined for this grid, e.g., the node in the lowest left corner of the grid can be considered as the origin, and based on the area, the orientation of the grid can be specified. By such definitions, the coordinates of the nodes are derived according to the grid resolution. The transmitter is located amongst these nodes in the localisation process; see [35,36]

Generally, for computing the VCMs of these nodes, the coordinates of the MSs are needed. The initial coordinates for these stations are specified in the defined local coordinate system. Therefore, many **A** matrices, created between the MSs and nodes, are computed solely from their coordinates, and therefore the VCMs from **A** matrices; see Equation (10). The total number of VCMs is equal to the number of grid nodes. Each VCM is a tool connecting the configuration, created between the transmitter and the MSs, to the quality of the estimated coordinates for the transmitter. This means that by varying the coordinates of the MSs the quality of the transmitter coordinates changes.

In a two-dimensional network, each VCM has four elements, two variances as diagonal and two equal covariances as off-diagonal. The desired VCMs or the square root of their traces (DOPs) for the nodes, can be regarded as two criteria for the optimisation of the network. In fact, the coordinates of the MSs vary until the estimated VCMs or DOPs are fitted to the desired ones.

In the following subsections, the mathematical models of these criteria and their relationship with the coordinates of the MSs are presented.

### 4.1. Variance-Covariance Matrix as a Criterion

When the VCM is selected for optimisation, its direct mathematical relation with the coordinates of the MSs is needed. To derive it, let us expand the VCM by the Taylor series for a node and four MSs of M, N, O and P (see [35,36,40,41,42,43]):

(12) $$C_{\hat{x}}={\left(A^{T}A\right)}^{-1}=C_{x}^{0}+\sum_{i=M,N,O,P}^{}\left[\begin{array}{cc} \frac{\partial C_{x}^{0}}{\partial x_{i}} & \frac{\partial C_{x}^{0}}{\partial y_{i}} \end{array}\right]\left[\begin{array}{l} \Delta x_{i}\\ \Delta y_{i} \end{array}\right]$$

where $C_{x}^{0}$ is the estimated VCM of the node of the initial design, $\Delta x_{i}$ and $\Delta y_{i}$ are the coordinate updates to the initial locations of the MSs, and $\frac{\partial C_{x}^{0}}{\partial x_{i}}$ and $\frac{\partial C_{x}^{0}}{\partial y_{i}}$ are, respectively, the partial derivatives of the VCM with respect to the coordinates of the *i*^th^ MS, with the following expressions (see [35,36]):

(13) $$\left\{\begin{array}{l} \frac{\partial C_{\hat{x}}^{0}}{\partial x_{i}}\\ \frac{\partial C_{\hat{x}}^{0}}{\partial y_{i}} \end{array}\right\}=-{\left(A^{T}A\right)}^{-1}\left(\left\{\begin{array}{l} \frac{\partial A^{T}}{\partial x_{i}}\\ \frac{\partial A^{T}}{\partial y_{i}} \end{array}\right\}A+A^{T}\left\{\begin{array}{l} \frac{\partial A}{\partial x_{i}}\\ \frac{\partial A}{\partial y_{i}} \end{array}\right\}\right){\left(A^{T}A\right)}^{-1}$$

where the elements of $\frac{\partial A}{\partial x_{i}}$ and $\frac{\partial A}{\partial y_{i}}$, are partial derivatives of **A** or the second-order partial derivatives of the observables. Note that the first-order derivative is taken with respect to the transmitter coordinates and the second-order one with respect to *x_i_* and *y_i_* -coordinates of the *i*^th^ MSs. The following general formulae are presented for the second-order partial derivatives of observable Equation (4) with respect to coordinates of *i*^th^ and *j*^th^ MSs:

(14) $$\frac{\partial^{2}\Omega_{ij}}{\partial x_{i}\partial x}=\frac{20\gamma }{\ln\left(10\right)d_{i}^{4}}\left({\left(x-x_{i}\right)}^{2}-\frac{d_{i}^{2}}{2}\right)\;\mathrm{and}\;\frac{\partial^{2}\Omega_{ij}}{\partial y_{i}\partial x}=\frac{20\gamma }{\ln\left(10\right)d_{i}^{4}}\left(y-y_{i}\right)\left(x-x_{i}\right)$$

(15) $$\frac{\partial^{2}\Omega_{ij}}{\partial x_{j}\partial x}=\frac{20\gamma }{\ln\left(10\right)d_{j}^{4}}\left(\frac{d_{j}^{2}}{2}-{\left(x-x_{j}\right)}^{2}\right)\;\mathrm{and}\;\frac{\partial \Omega_{ij}}{\partial y_{j}\partial x}=-\frac{20\gamma }{\ln\left(10\right)d_{j}^{4}}\left(y-y_{j}\right)\left(x-x_{j}\right)$$

(16) $$\frac{\partial^{2}\Omega_{ij}}{\partial y_{i}\partial y}=\frac{20\gamma }{\ln\left(10\right)d_{i}^{4}}\left({\left(y-y_{i}\right)}^{2}-\frac{d_{i}^{2}}{2}\right)\;\mathrm{and}\;\frac{\partial^{2}\Omega_{ij}}{\partial x_{i}\partial y}=\frac{20\gamma }{\ln\left(10\right)d_{i}^{4}}\left(y-y_{i}\right)\left(x-x_{i}\right)$$

(17) $$\frac{\partial^{2}\Omega_{ij}}{\partial y_{j}\partial x}=\frac{20\gamma }{\ln\left(10\right)d_{j}^{4}}\left(\frac{d_{j}^{2}}{2}-{\left(y-y_{j}\right)}^{2}\right)\;\mathrm{and}\;\frac{\partial \Omega_{ij}}{\partial y_{j}\partial y}=-\frac{20\gamma }{\ln\left(10\right)d_{j}^{4}}\left(y-y_{j}\right)\left(x-x_{j}\right)$$

From these general formulae, structures of the derivatives of **A** with respect to coordinates of M, N, O and P, are:

(18) $$\frac{\partial A}{\partial x_{M}}=\frac{20\gamma }{\ln\left(10\right)d_{M}^{4}}\left[\begin{array}{cc} {\left(x-x_{M}\right)}^{2}-\frac{d_{M}^{2}}{2} & \left(x-x_{M}\right)\left(y-y_{M}\right)\\ {\left(x-x_{M}\right)}^{2}-\frac{d_{M}^{2}}{2} & \left(x-x_{M}\right)\left(y-y_{M}\right)\\ {\left(x-x_{M}\right)}^{2}-\frac{d_{M}^{2}}{2} & \left(x-x_{M}\right)\left(y-y_{M}\right)\\ 0 & 0\\ 0 & 0\\ 0 & 0 \end{array}\right]\frac{\partial A}{\partial y_{M}}=\frac{20\gamma }{\ln\left(10\right)d_{M}^{4}}\left[\begin{array}{cc} \left(y-y_{M}\right)\left(x-x_{M}\right) & {\left(y-y_{M}\right)}^{2}-\frac{d_{M}^{2}}{2}\\ \left(y-y_{M}\right)\left(x-x_{M}\right) & {\left(y-y_{M}\right)}^{2}-\frac{d_{M}^{2}}{2}\\ \left(y-y_{M}\right)\left(x-x_{M}\right) & {\left(y-y_{M}\right)}^{2}-\frac{d_{M}^{2}}{2}\\ 0 & 0\\ 0 & 0\\ 0 & 0 \end{array}\right]$$

(19) $$\frac{\partial A}{\partial x_{N}}=\frac{20\gamma }{\ln\left(10\right)d_{N}^{4}}\left[\begin{array}{cc} \frac{d_{N}^{2}}{2}-{\left(x-x_{N}\right)}^{2} & \left(x-x_{N}\right)\left(y-y_{N}\right)\\ 0 & 0\\ 0 & 0\\ {\left(x-x_{N}\right)}^{2}-\frac{d_{N}^{2}}{2} & \left(x-x_{N}\right)\left(y-y_{N}\right)\\ {\left(x-x_{N}\right)}^{2}-\frac{d_{N}^{2}}{2} & \left(x-x_{N}\right)\left(y-y_{N}\right)\\ 0 & 0 \end{array}\right],\;\frac{\partial A}{\partial y_{N}}=\frac{20\gamma }{\ln\left(10\right)d_{N}^{4}}\left[\begin{array}{cc} \left(x-x_{N}\right)\left(y-y_{N}\right) & \frac{d_{N}^{2}}{2}-{\left(y-y_{N}\right)}^{2}\\ 0 & 0\\ 0 & 0\\ \left(x-x_{N}\right)\left(y-y_{N}\right) & {\left(y-y_{N}\right)}^{2}-\frac{d_{N}^{2}}{2}\\ \left(x-x_{N}\right)\left(y-y_{N}\right) & {\left(y-y_{N}\right)}^{2}-\frac{d_{N}^{2}}{2}\\ 0 & 0 \end{array}\right]$$

(20) $$\frac{\partial A}{\partial x_{O}}=\frac{20\gamma }{\ln\left(10\right)d_{O}^{4}}\left[\begin{array}{cc} 0 & 0\\ \frac{d_{O}^{2}}{2}-{\left(x-x_{O}\right)}^{2} & \left(x-x_{O}\right)\left(y-y_{O}\right)\\ 0 & 0\\ \frac{d_{O}^{2}}{2}-{\left(x-x_{O}\right)}^{2} & \left(x-x_{O}\right)\left(y-y_{O}\right)\\ 0 & 0\\ {\left(x-x_{O}\right)}^{2}-\frac{d_{O}^{2}}{2} & \left(x-x_{O}\right)\left(y-y_{O}\right) \end{array}\right],\;\frac{\partial A}{\partial y_{O}}=\frac{20\gamma }{\ln\left(10\right)d_{O}^{4}}\left[\begin{array}{cc} 0 & 0\\ \left(x-x_{O}\right)\left(y-y_{O}\right) & \frac{d_{O}^{2}}{2}-{\left(y-y_{O}\right)}^{2}\\ 0 & 0\\ \left(x-x_{O}\right)\left(y-y_{O}\right) & \frac{d_{O}^{2}}{2}-{\left(y-y_{O}\right)}^{2}\\ 0 & 0\\ \left(x-x_{O}\right)\left(y-y_{O}\right) & {\left(y-y_{O}\right)}^{2}-\frac{d_{O}^{2}}{2} \end{array}\right]$$

(21) $$\frac{\partial A}{\partial x_{P}}=\frac{20\gamma }{\ln\left(10\right)d_{P}^{4}}\left[\begin{array}{cc} 0 & 0\\ 0 & 0\\ \frac{d_{P}^{2}}{2}-{\left(x-x_{P}\right)}^{2} & \left(x-x_{P}\right)\left(y-y_{P}\right)\\ 0 & 0\\ \frac{d_{P}^{2}}{2}-{\left(x-x_{P}\right)}^{2} & \left(x-x_{P}\right)\left(y-y_{P}\right)\\ \frac{d_{P}^{2}}{2}-{\left(x-x_{P}\right)}^{2} & \left(x-x_{P}\right)\left(y-y_{P}\right) \end{array}\right],\;\frac{\partial A}{\partial y_{P}}=\frac{20\gamma }{\ln\left(10\right)d_{P}^{4}}\left[\begin{array}{cc} 0 & 0\\ 0 & 0\\ \left(x-x_{P}\right)\left(y-y_{P}\right) & \frac{d_{P}^{2}}{2}-{\left(y-y_{P}\right)}^{2}\\ 0 & 0\\ \left(x-x_{P}\right)\left(y-y_{P}\right) & \frac{d_{P}^{2}}{2}-{\left(y-y_{P}\right)}^{2}\\ \left(x-x_{P}\right)\left(y-y_{P}\right) & \frac{d_{P}^{2}}{2}-{\left(y-y_{P}\right)}^{2} \end{array}\right].$$

### 4.2. DOP as a Criterion

DOP is the square root of the trace of the VCM of a node and its Taylor expansion with respect to the coordinates of the MSs is:

(22) $$DOP=\sqrt{\mathrm{trace}\left(C_{x}^{0}\right)}+\sum_{i=M,N,O,P}^{}\left[\begin{array}{cc} \frac{\partial \sqrt{\mathrm{trace}\left(C_{x}^{0}\right)}}{\partial x_{i}} & \frac{\partial \sqrt{\mathrm{trace}\left(C_{x}^{0}\right)}}{\partial y_{i}} \end{array}\right]\left[\begin{array}{l} \Delta x_{i}\\ \Delta y_{i} \end{array}\right]$$

where trace() stands for the trace operator or the sum of the diagonal elements of a square matrix, $\frac{\partial \sqrt{\mathrm{trace}\left(C_{x}^{0}\right)}}{\partial x_{i}}$ and $\frac{\partial \sqrt{\mathrm{trace}\left(C_{x}^{0}\right)}}{\partial y_{i}}$ are respectively partial derivatives of DOP with respect to the coordinates of the *i*^th^ MS with the following formula:

(23) $$\left\{\begin{array}{l} \frac{\partial DOP}{\partial x_{i}}\\ \frac{\partial DOP}{\partial y_{i}} \end{array}\right\}=-\frac{1}{2}\mathrm{trace}\left({\left(A^{T}A\right)}^{-1}\left(\left\{\begin{array}{l} \frac{\partial A^{T}}{\partial x_{i}}\\ \frac{\partial A^{T}}{\partial y_{i}} \end{array}\right\}A+A^{T}\left\{\begin{array}{l} \frac{\partial A}{\partial x_{i}}\\ \frac{\partial A}{\partial y_{i}} \end{array}\right\}\right){\left(A^{T}A\right)}^{-1}\right){\left(\mathrm{trace}\left(C_{x}^{0}\right)\right)}^{-\frac{1}{2}}$$

The structures of $\frac{\partial A}{\partial y_{i}}$ and $\frac{\partial A}{\partial x_{i}}$ have been already discussed in the previous section.

## 5. Optimisation Model and Required Constraints

The expanded VCM and DOP by the Taylor series, Equations (12) and (22), are our mathematical models for optimal estimation of updates to the coordinates of the MSs. On the left-hand side of these equations, the desired VCMs and DOPs are, and their right-hand sides are computed from the coordinates of a node and the initial coordinates of the MSs. The estimated initial VCMs and DOPs do not have a good fit for the desired ones, therefore, by changing the coordinates of the MSs using quadratic optimisation, we try to fit them.

It is important to note that the number of unknowns, or the coordinate updates, depends on the number of the MSs. Since four MSs are used in this study, then, the number of unknowns becomes eight as the network is two-dimensional. Each VCM has four elements meaning that four equations are created for one node. Such a system is under-determined, and more equations are needed to solve the system. By adding the VCM of another node, the number of equations increases by four. Consequently, considering each extra node leads to four additional equations in the system. As mentioned before, a grid of nodes, covering the area, is designed in the earliest stage where the resolution and size of the area specify the number of nodes. In short, there are many nodes over the area and the number of equations is four times larger than the number of nodes. Therefore, there is enough redundancy for optimisation.

The situation is rather similar for optimisation based on DOP with only one difference. For each node, only one DOP is defined, and the number of equations is equal to the number of nodes. The system of equations is not as redundant as that one based on the VCM, but both have enough redundancies.

Let us present the overdetermined system in the following Gauss-Markov form:

(24) $$B_{k}\Delta x=\Delta L_{k}-\varepsilon_{k}\;\mathrm{where}\;k=\mathrm{VCM}\;\mathrm{or}\;\mathrm{DOP}$$

where Δx stands for the MSs’ coordinate updates

(25) $$\Delta x={\left[\begin{array}{ccccccc} \Delta x_{M} & \Delta y_{M} & \Delta x_{N} & \Delta y_{N} & \begin{array}{cc} \Delta x_{O} & \Delta y_{O} \end{array} & \Delta x_{P} & \Delta y_{P} \end{array}\right]}^{T}$$

$\varepsilon_{k}$ is the vector of residuals, and $\Delta L_{k}$ the vector of differences between the elements of the criterion and initial VCMs or their DOPs, $B_{k}$ stands for the coefficient’s matrix containing the partial derivatives of the VCM, or DOP with respect to the MSs’ coordinates. In the case of using the VCM for optimisation, we have

(26) $$\Delta L_{\mathrm{VCM}}={\left[\begin{array}{cccc} \mathrm{vec}\left(C_{{\hat{x}}_{1}}\right)-\mathrm{vec}\left(C_{x_{1}}^{0}\right) & \mathrm{vec}\left(C_{{\hat{x}}_{2}}\right)-\mathrm{vec}\left(C_{x_{2}}^{0}\right) & \cdots & \mathrm{vec}\left(C_{{\hat{x}}_{n}}\right)-\mathrm{vec}\left(C_{x_{n}}^{0}\right) \end{array}\right]}^{T}$$

(27) $$B_{\mathrm{VCM}}={\left[\begin{array}{ccccccc} \mathrm{vec}\left(\frac{\partial C_{x_{1}}^{0}}{\partial x_{M}}\right) & \mathrm{vec}\left(\frac{\partial C_{x_{1}}^{0}}{\partial y_{M}}\right) & \mathrm{vec}\left(\frac{\partial C_{x_{1}}^{0}}{\partial x_{N}}\right) & \mathrm{vec}\left(\frac{\partial C_{x_{1}}^{0}}{\partial y_{N}}\right) & \begin{array}{cc} \mathrm{vec}\left(\frac{\partial C_{x_{1}}^{0}}{\partial y_{O}}\right) & \mathrm{vec}\left(\frac{\partial C_{x_{1}}^{0}}{\partial y_{O}}\right) \end{array} & \mathrm{vec}\left(\frac{\partial C_{x_{1}}^{0}}{\partial x_{P}}\right) & \mathrm{vec}\left(\frac{\partial C_{x_{1}}^{0}}{\partial y_{P}}\right)\\ \mathrm{vec}\left(\frac{\partial C_{x_{2}}^{0}}{\partial x_{M}}\right) & \mathrm{vec}\left(\frac{\partial C_{x_{2}}^{0}}{\partial y_{M}}\right) & \mathrm{vec}\left(\frac{\partial C_{x_{2}}^{0}}{\partial x_{N}}\right) & \mathrm{vec}\left(\frac{\partial C_{x_{2}}^{0}}{\partial y_{N}}\right) & \begin{array}{cc} \mathrm{vec}\left(\frac{\partial C_{x_{2}}^{0}}{\partial y_{O}}\right) & \mathrm{vec}\left(\frac{\partial C_{x_{2}}^{0}}{\partial y_{O}}\right) \end{array} & \mathrm{vec}\left(\frac{\partial C_{x_{2}}^{0}}{\partial x_{P}}\right) & \mathrm{vec}\left(\frac{\partial C_{x_{2}}^{0}}{\partial y_{P}}\right)\\ \vdots & \vdots & \vdots & \vdots & \vdots & \vdots & \vdots \\ \mathrm{vec}\left(\frac{\partial C_{x_{n}}^{0}}{\partial x_{M}}\right) & \mathrm{vec}\left(\frac{\partial C_{x_{n}}^{0}}{\partial y_{M}}\right) & \mathrm{vec}\left(\frac{\partial C_{x_{n}}^{0}}{\partial x_{N}}\right) & \mathrm{vec}\left(\frac{\partial C_{x_{n}}^{0}}{\partial y_{N}}\right) & \begin{array}{cc} \mathrm{vec}\left(\frac{\partial C_{x_{n}}^{0}}{\partial y_{O}}\right) & \mathrm{vec}\left(\frac{\partial C_{x_{n}}^{0}}{\partial y_{O}}\right) \end{array} & \mathrm{vec}\left(\frac{\partial C_{x_{n}}^{0}}{\partial x_{P}}\right) & \mathrm{vec}\left(\frac{\partial C_{x_{n}}^{0}}{\partial y_{P}}\right) \end{array}\right]}_{4n\times 8}$$

where operator “vec” inserts the columns of the VCM below each other and converts the 2 × 2 matrices to 4 × 1 vectors, *n* means the number of the nodes.

When the DOP is used for optimisation, we have:

(28) $$\Delta L_{\mathrm{DOP}}={\left[\begin{array}{cccc} \mathrm{trace}\left(C_{{\hat{x}}_{1}}\right)-\mathrm{trace}\left(C_{x_{1}}^{0}\right) & \mathrm{trace}\left(C_{{\hat{x}}_{2}}\right)-\mathrm{trace}\left(C_{x_{2}}^{0}\right) & \cdots & \mathrm{trace}\left(C_{{\hat{x}}_{n}}\right)-\mathrm{trace}\left(C_{x_{n}}^{0}\right) \end{array}\right]}^{T}$$

(29) $$B_{\mathrm{DOP}}={\left[\begin{array}{ccccccc} \frac{\partial \mathrm{trace}\left(C_{x_{1}}^{0}\right)}{\partial x_{M}} & \frac{\partial \mathrm{trace}\left(C_{x_{1}}^{0}\right)}{\partial y_{M}} & \frac{\partial \mathrm{trace}\left(C_{x_{1}}^{0}\right)}{\partial x_{N}} & \frac{\partial \mathrm{trace}\left(C_{x_{1}}^{0}\right)}{\partial y_{N}} & \begin{array}{cc} \frac{\partial \mathrm{trace}\left(C_{x_{1}}^{0}\right)}{\partial y_{O}} & \frac{\partial \mathrm{trace}\left(C_{x_{1}}^{0}\right)}{\partial y_{O}} \end{array} & \frac{\partial \mathrm{trace}\left(C_{x_{1}}^{0}\right)}{\partial x_{P}} & \frac{\partial \mathrm{trace}\left(C_{x_{1}}^{0}\right)}{\partial y_{P}}\\ \frac{\partial \mathrm{trace}\left(C_{x_{2}}^{0}\right)}{\partial x_{M}} & \frac{\partial \mathrm{trace}\left(C_{x_{2}}^{0}\right)}{\partial y_{M}} & \frac{\partial \mathrm{trace}\left(C_{x_{2}}^{0}\right)}{\partial x_{N}} & \frac{\partial \mathrm{trace}\left(C_{x_{2}}^{0}\right)}{\partial y_{N}} & \begin{array}{cc} \frac{\partial \mathrm{trace}\left(C_{x_{2}}^{0}\right)}{\partial y_{O}} & \frac{\partial \mathrm{trace}\left(C_{x_{2}}^{0}\right)}{\partial y_{O}} \end{array} & \frac{\partial \mathrm{trace}\left(C_{x_{2}}^{0}\right)}{\partial x_{P}} & \frac{\partial \mathrm{trace}\left(C_{x_{2}}^{0}\right)}{\partial y_{P}}\\ \vdots & \vdots & \vdots & \vdots & \vdots & \vdots & \vdots \\ \frac{\partial \mathrm{trace}\left(C_{x_{n}}^{0}\right)}{\partial x_{M}} & \frac{\partial \mathrm{trace}\left(C_{x_{n}}^{0}\right)}{\partial y_{M}} & \frac{\partial \mathrm{trace}\left(C_{x_{n}}^{0}\right)}{\partial x_{N}} & \frac{\partial \mathrm{trace}\left(C_{x_{n}}^{0}\right)}{\partial y_{N}} & \begin{array}{cc} \frac{\partial \mathrm{trace}\left(C_{x_{n}}^{0}\right)}{\partial y_{O}} & \frac{\partial \mathrm{trace}\left(C_{x_{n}}^{0}\right)}{\partial y_{O}} \end{array} & \frac{\partial \mathrm{trace}\left(C_{x_{n}}^{0}\right)}{\partial x_{P}} & \frac{\partial \mathrm{trace}\left(C_{x_{n}}^{0}\right)}{\partial y_{P}} \end{array}\right]}_{n\times 8}$$

The system of Equation (24) should be solved for the coordinate updates in a least-squares sense, but Eshagh [35] has mentioned that applying some constraints in the quadratic optimisation model is necessary, otherwise, instability and improper configurations are obtained. Therefore, the following optimisation model for solving the system (24) is suggested:

(30) $$\min\left(\frac{1}{2}\Delta x^{T}B_{i}^{T}B_{i}^{}\Delta x-B_{i}^{T}\Delta L_{i}^{}\right)\;\mathrm{where}\;i=\mathrm{VCM}\;\mathrm{or}\;\mathrm{DOP}\;$$

Subject to

(31) DΔx=d

(32) $$L_{b}\leq \Delta x\leq U_{b}$$

The first constraint (31) controls the coordinate updates in such a way that the MSs move towards some specified directions. The mathematical derivations of them are available in [36]. **D** and **d** have the following structures:

(33) $$D=\left[\begin{array}{cccccccc} 1 & -{\mathrm{tanAz}}_{MM^{\prime }} & 0 & 0 & 0 & 0 & 0 & 0\\ 0 & 0 & 1 & -{\mathrm{tanAz}}_{NN^{\prime }} & 0 & 0 & 0 & 0\\ 0 & 0 & 0 & 0 & 1 & -{\mathrm{tanAz}}_{OO^{\prime }} & 0 & 0\\ 0 & 0 & 0 & 0 & 0 & 0 & 1 & -{\mathrm{tanAz}}_{PP^{\prime }} \end{array}\right]$$

(34) $$d=0-\left[\begin{array}{c} x_{M}-x_{M^{\prime }}-{\mathrm{tanAz}}_{M^{\prime }M}\left(y_{M}-y_{M^{\prime }}\right)\\ x_{N}-x_{N^{\prime }}-{\mathrm{tanAz}}_{N^{\prime }N}\left(y_{N}-y_{N^{\prime }}\right)\\ x_{O}-x_{O^{\prime }}-{\mathrm{tanAz}}_{O^{\prime }O}\left(y_{O}-y_{O^{\prime }}\right)\\ x_{P}-x_{P^{\prime }}-{\mathrm{tanAz}}_{P^{\prime }P}\left(y_{P^{\prime }}-y_{P^{\prime }}\right) \end{array}\right]$$

where Az stands for the specified azimuth in which the MSs should move during the optimisation process. M’, N’, O’ and P’ are, respectively, some helpful points that M, N, O and P should move towards them.

The second constraint (32) define the search area around each MS, and the coordinate updates are computed in such a way that the MSs remain inside the search area; for details see [35,36]:

(35) $$L_{b}={\left[\begin{array}{ccccccc} w_{M}^{L}-x_{M} & v_{M}^{L}-y_{M} & w_{N}^{L}-x_{N} & v_{N}^{L}-y_{N} & \begin{array}{cc} w_{O}^{L}-x_{O} & v_{O}^{L}-y_{O} \end{array} & w_{P}^{L}-x_{P} & v_{P}^{L}-y_{P} \end{array}\right]}^{T}$$

(36) $$U_{b}={\left[\begin{array}{ccccccc} w_{M}^{U}-x_{M} & v_{M}^{U}-y_{M} & w_{N}^{U}-x_{N} & v_{N}^{U}-y_{N} & \begin{array}{cc} w_{O}^{U}-x_{O} & v_{O}^{U}-y_{O} \end{array} & w_{P}^{U}-x_{P} & v_{P}^{U}-y_{P} \end{array}\right]}^{T}$$

$w_{i}^{L}$ and $w_{i}^{U}$, *i* = M, N, O and P, are, respectively, the lower and upper bounds of the search area for the *x*-coordinate of the *i*^th^ MS, and $v_{i}^{L}$ and $v_{i}^{U}$, are similar ones for the *y*-coordinate.

## 6. Design of Interference Localisation Security Network for the Arlanda International Airport Based on Received Signal Strength Differences

For testing our methodology, we select the Arland international airport of Sweden, in the northern part of Stockholm. The airport is almost square in shape and has three runways. The goal is to optimally design a four-MS interference localisation network on the runways. The VCM and DOP criteria are defined and applied to optimise the geometric configuration of MSs. After that the resolution and precision are presented and discussed.

**Figure 1:**
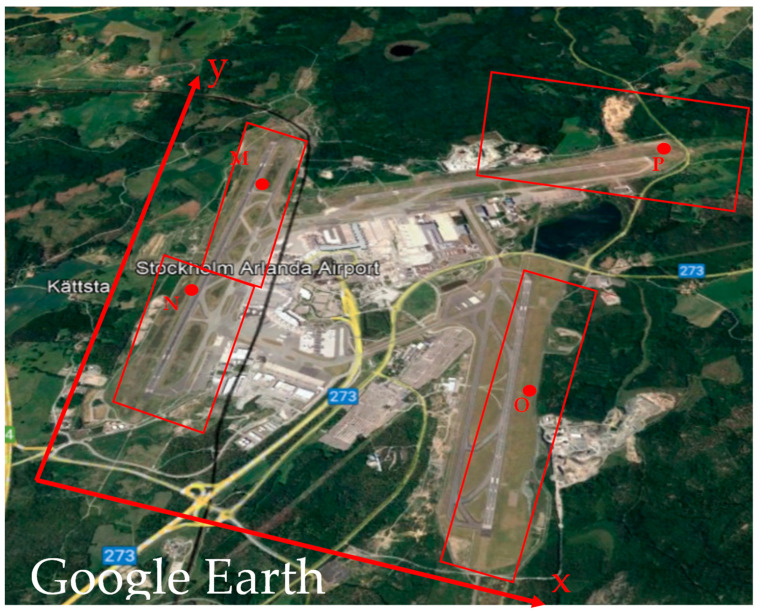
The local coordinate system, monitoring stations (MSs) and search areas of the MSs on the satellite photo, taken from Google Earth, of the Arlanda international airport of Sweden.

We consider rectangular search areas around each MS; see Figure 1. As the figure shows a two-dimensional coordinates system is defined with an origin outside the airport in the south-west part of the area with the geodetic coordinates of φ = 59°37′10′′ and λ = 17°53′50′′. The *y*-axis is parallel to the western runway having an azimuth of 10°, and the *x*-axis is perpendicular to the *y*-axis with an azimuth of 100°.

The updates to the *x*-coordinates of M, N and O are estimated in such a way that these points move ±250 m from their initial values according to the width of the runways. *y*_M_, *y*_N_ and *y*_O_ are limited, respectively, between −2000 to 500 m, +1000 and −2000 m, +1000 and −2000 m. *x*_P_ is limited between −1000 and 1000 m, and *y*_P_ between −500 and 500 m. Figure 1 shows the photo of the Arlanda international airport (taken from Google Earth), the local coordinate system, and the initial positions of M, N, O, P, shown by the small red circles and their rectangular search areas. Our goal is to keep the MSs on the runways during the optimal design procedure.

In addition, as observed the *y*-axis of the system is chosen parallel to the western runway for simplification. However, the choice of the coordinate system is not important as the design can be conducted based on any chosen system and later the whole network is georeferenced. Since the eastern and western runways have an azimuth of about 10° then the *y*-axis of the system has the same azimuth.

Table 1 shows the coordinates of the MSs before and after optimisation based on the VCM and DOP criteria for *γ* = 2, 4 and 6. As mentioned before, *γ* can be estimated in the calibration step of localisation. However, since there is no measurement in the design step, then we consider these three values to test the role of *γ* in the design. *γ* is shown in the left most column of the table, and in the column before, the criteria of VCM and DOP are specified. Three resolutions of 20, 40 and 80 m are considered to check their effects on the design.

As seen in Table 1, *x*_M_, *x*_N_ and *x*_O_ do not change by the resolutions, criteria and *γ* because M, N and O are in the northward runways of the airport, almost parallel to the *y*-axis of the defined coordinate system. The directional constraints (31) keep the movements of the MSs along these runways, therefore, it is normal to see no change in their *x*-coordinates but large changes in the *y*-coordinates.

From Table 1, based on both the VCM and DOP criteria, the optimised coordinates do not show any sensitivity to the choice of *γ* for the resolutions 40 and 80 m. For the resolution 20 m with the DOP criterion the optimal coordinates are not significantly different for the selected pathloss exponents. For the VCM criterion, the optimal results are the same for *γ =* 4 and 6.

Generally, we can conclude that lower resolutions than 40 m and *γ* have no significant influence on the optimal coordinates. For the resolution 20 m and higher in the free space γ = 2, the results are different.

Table 2 illustrates the statistics of the DOP values before and after optimisation for *γ* = 2, 4 and 6 and the resolutions of 10, 20 and 40 m. The chosen value for *γ* has a significant influence on the DOP of the network. Convergence is important in any optimisation process and in the optimisation of a security network for localisation, having a convergent solution is not straightforward. In addition, expecting convergence for any selected values for the VCM or DOP criterion is neither logical nor realistic. For example, selecting the minimum DOP of the initial design is not realistic and the optimisation process diverges. The statistics of the initial DOPs are indicators of the right choice of criterion values. In our study, selecting the mean value of the initial DOP and optimising the network based on that in all three values of *γ* leads to convergence. According to Table 2, when *γ* = 2, the mean DOP is 4.8, this means that the network is optimised by changing the positions of the MSs in such a way that the DOPs are fitted to 4.8. The mean DOPs are 2.4 and 1.6, respectively, for *γ* = 4 and 6. A diagonal matrix with diagonal elements equal to the square of the mean DOP is considered the VCM criterion for all nodes.

The VCM criterion, as shown in Section 4.1, is the expected VCM for all points, and the DOP criterion is the square root of a trace of this VCM. As Table 2 shows, the statistics are almost the same for both chosen criteria, the reason is that in both cases the DOP of the network is presented. However, when the DOP criterion is used, there is no control over the covariances between the estimated coordinates. In addition, for each point, four equations are created in the system when the VCM criterion is applied but only one equation when the DOP criterion is utilised. Therefore, the VCM criterion adds a larger number of equations, considers covariances, and has the possibility of selecting a special structure for the VCM. For example, two different values for the variances of the *x*- and *y*-coordinates can be selected, as well as any correlation between coordinates, if required.

The numerical optimisation process is time-consuming and has low rates of convergence for high-resolution grids. In other words, the process needs a large number of iterations to converge. In this study, when the norm of the coordinate updates becomes less than 1 m, the iteration is stopped. Considering smaller values is also possible but with the costs of many iterations and lower convergence rate.

**Figure 2:**
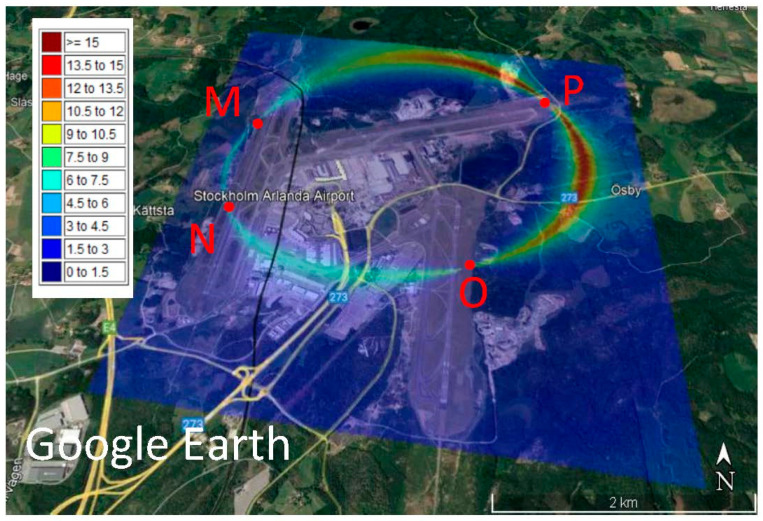
Dilution of precision (DOP) of the initial design of a security localisation network based on the received signal strength differences (RSSD) over the Google Earth map of the Arlanda international airport.

By assuming that the Arlanda international airport is flat, we select γ = 4. Figure 2 is the map of the initial DOPs of the network with the locations of the MSs M, N, O and P. The maximum DOP reaches 15, and the large values are along an ellipse form going through the MSs. Most of these large values are outside the airport and in the surrounding forests, except those between stations N and O.

**Figure 3:**
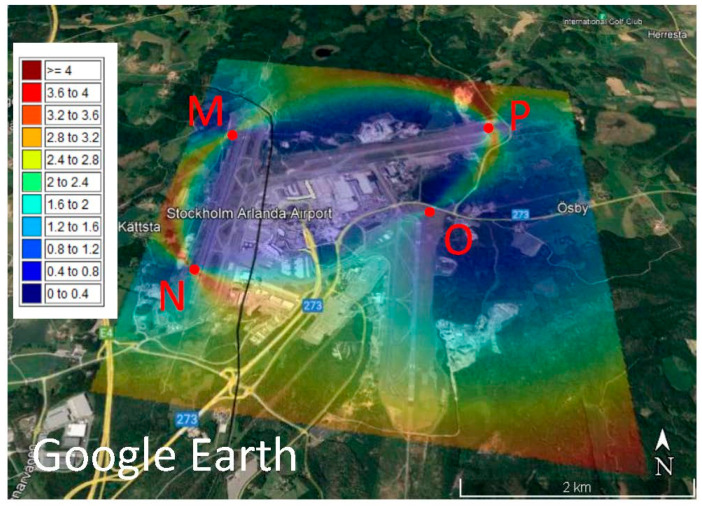
Dilution of precision (DOP) of the optimal design of a security localisation network with the variance-covariance matrix (VCM) criterion based on the received signal strength differences (RSSD) over the Google Earth map of the Arlanda international airport.

Figure 3 is the map of the DOPs after optimisation of the network based on the VCM criterion for the Arlanda international airport. A significant reduction is observed in the DOP values and as Table 2 shows, the maximum reaches 4. The stations M moved slightly northward and N southward and both along the specified azimuth of 10°. Point O is located at the northern part of the runway and P with slight displacement along the runway. As observed, the high values are in the form of an ellipse passing through the MSs, this ellipse is smaller than the one created based on the initial design. Some large DOPs are seen in the southeast of the area, but they are less than 4.

**Figure 4:**
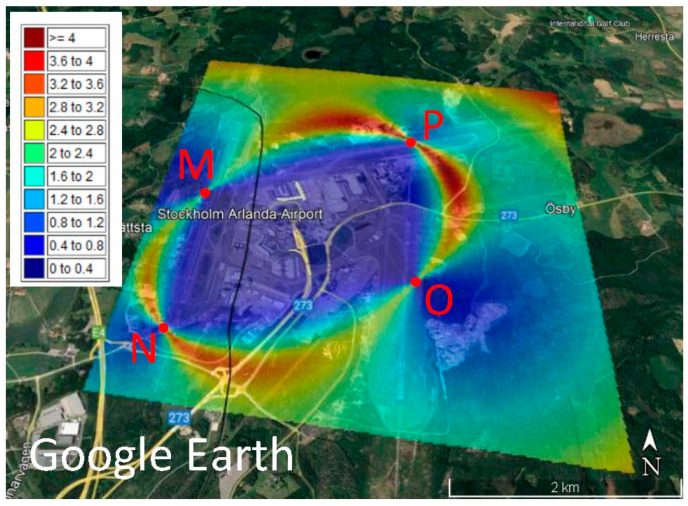
Dilution of precision (DOP) of the optimal design of a security localisation network with the DOP criterion based on the received signal strength differences (RSSD) over the Google Earth map of the Arlanda international airport.

Figure 4 is the map of DOPs after optimisation based on the DOP criterion. Again, large values are seen along an ellipse passing through the MSs. The DOP values are significantly reduced so that their maximum does not exceed 4. Station M is in the northern part of the runway, and N is moved to the most southern part of it. P and Q are found closer to the middle of their runways.

A comparison of Figure 2, which is the map of the DOP of the initial design, and Figure 3 and Figure 4, shows that the optimisation process is successful, because a maximum DOP of 15 in the initial design reduces to 4 in both optimal designs. Figure 2, Figure 3 and Figure 4 show ellipse forms for the large DOPs passing through the four MSs, but with different sizes and orientations because the optimal positions of the MSs are not the same. Figure 2 shows large DOPs, reaching 15, from the station P towards Q and M, and smaller values, about 6, between M and N; see Figure 2, but after optimisation using the VCM criterion, these values decrease to 4 and even smaller around Q; see Figure 3. However, Figure 4 shows values around 4 amongst all MSs and the map is more symmetric compared to the map presented in Figure 3. Interestingly, Figure 4 shows small values of DOP over the buildings in the area unlike Figure 2 and Figure 3, but this is accidental. If the goal of optimisation is to have small DOPs over buildings, the optimisation criterion can be designed specifically for this purpose.

## 7. Concluding Remarks

In this paper, the received signal strength differences (RSSDs) were used as observables and a localisation network was optimally designed based on the variance-covariance matrix (VCM) and dilution of precision (DOP) criteria. All mathematical derivations and formulae required for optimal design based on the RSSDs, these criteria, and the required constraints were developed and successfully applied for designing an optimal wireless localisation network for the Arlanda international airport of Sweden. The pathloss exponent (*γ*) of the RSSDs showed no significant effect on the final optimal coordinates of the monitoring stations (MSs) for lower resolutions than 20 × 20 m. In addition, no significant difference was observed in these optimal coordinates based on the VCM and DOP criteria and applying the same directional and search area constraints. For the case of using γ = 2, considering localisation in a free space, and higher resolution different optimal coordinates were observed. The statistics of the DOPs after optimisation for different resolutions and criteria were almost the same for each, but not their maps. These maps were different over the Arlanda international airport for γ = 4, even if the optimal coordinates are the same. This could be because of the higher redundancy of the system and considering zero covariances when the VCM criterion was applied. This criterion was more suitable because of higher redundancy in the optimisation, and flexibility of considering more information. The correlations amongst the RSSDs and different values for variances for *x*- and *y*-coordinates could be counted but such a possibility did not exist for the DOP criterion.

## Figures and Tables

**Table 1:** Coordinates of the monitoring stations (MSs) in metres before and after optimisation with different resolutions, pathloss exponents and the VCM and DOP criteria [m].

			$x_{M}$	$y_{M}$	$x_{N}$	$y_{N}$	$x_{O}$	$y_{O}$	$x_{P}$	$y_{P}$
			550.5	4000.5	550.5	2500.5	3099.5	1999.5	3999.5	4999.5
γ = 2	VCM	20 m	550.3	4411.7	550.5	1867.3	3099.5	3209.3	4019.2	5008.9
		40 m	550.3	4112	550.5	1365	3099.5	2995.5	3919.8	4960.4
		80 m	550.3	3481.8	550.5	883.6	3099.5	2445.3	3437.9	4725.4
	DOP	20 m	550.3	3051.8	550.5	912.3	3099.5	1998.5	2999.5	4511.6
		40 m	550.3	3099.2	550.5	1013.2	3099.5	2055.2	2999.5	4511.6
		80 m	550.3	3416.9	550.5	1137.5	3099.5	2272	3276.6	4646.7
γ = 4	VCM	20 m	550.3	4272.6	550.5	1747.5	3099.5	3145.8	3920.2	4960.6
		40 m	550.3	4112	550.5	1365	3099.5	2995.6	3920.1	4960.6
		80 m	550.3	3481.8	550.5	883.6	3099.5	2445.3	3437.9	4725.4
	DOP	20 m	550.3	4272.6	550.5	1747.1	3099.5	3145.8	3920.2	4960.6
		40 m	550.3	3099.2	550.5	1013.3	3099.5	2055.3	2999.5	4511.6
		80 m	550.3	3416.9	550.5	1137.5	3099.5	2272	3276.6	4646.7
γ = 6	VCM	20 m	550.3	4272.5	550.5	1747.1	3099.5	3145.6	3920.2	4960.6
		40 m	550.3	4112	550.5	1365	3099.5	2995.6	3920.1	4960.6
		80 m	550.3	3481.8	550.5	883.6	3099.5	2445.3	3437.9	4725.4
	DOP	20 m	550.3	3051.7	550.5	912.2	3099.5	1998	2999.5	4511.6
		40 m	550.3	3099.1	550.5	1013.1	3099.5	2055.2	2999.5	4511.6
		80 m	550.3	3416.9	550.5	1137.5	3099.5	2272	3276.6	4646.7

**Table 2:** Dilution of precision (DOP) before and after optimisation based on the VCM and DOP criteria and different values of γ.

		VCM Criterion				DOP Criterion			
		Min	Mean	Max	Std	Min	Mean	Max	Std
γ = 2	Initial	0.7	4.8	29.3	4.4	0.7	4.8	29.3	4.4
	20 m	0.9	3.4	8.2	1.7	0.9	3.4	8.0	1.6
	40 m	0.9	3.3	7.5	1.6	0.9	3.4	8.1	1.6
	80 m	1	3.3	7.0	1.5	0.9	3.3	7.8	1.6
γ = 4	Initial	0.4	2.4	14.7	2.2	0.4	2.4	14.7	2.2
	20 m	0.4	1.7	4.1	0.8	0.4	1.7	4.0	0.8
	40 m	0.5	1.6	3.7	0.8	0.4	1.7	4.1	0.8
	80 m	0.5	1.6	3.5	0.8	0.5	1.7	3.9	0.8
γ = 6	Initial	0.2	1.6	9.8	1.5	0.2	1.6	9.8	1.5
	20 m	0.3	1.1	2.7	0.6	0.3	1.1	2.7	0.5
	40 m	0.3	1.1	2.5	0.5	0.3	1.1	2.7	0.5
	80 m	0.3	1.1	2.3	0.5	0.3	1.1	2.6	0.5

## Data Availability

Not applicable.

## References

[1] Dempster A. (2016). Interference localization from satellite navigation systems. Proc. IEEE.

[2] Humphreys T.E., Ledvina B.M., Psiaki M.L., O’Hanlon B.W., Kintner P.M. Assessing the spoofing threat: Development of a portable GPS civilian spoofer. Proceedings of the 21st International Technical Meeting of the Satellite Division of the Institute of Navigation (ION GNSS 2008).

[3] Divis D.A. (2013). GPS Spoofing Experiment Knocks Ship Off Course.

[4] Clynch J.R., Parker A.A., Adler R.W., Vincent W.R. (2003). System Challenge—The Hunt for RFI—Unjamming a Coast Harbor.

[5] Balaei A.T., Motella B., Dempster A.G. GPS interference detected in Sydney Australia. Proceedings of the IGNSS Conference.

[6] Motella B., Pini M., Dovis F. (2008). Investigation on the effect of strong out-of-band signals on global navigation satellite systems receivers. GPS Solut..

[7] Grant A., Williams P., Ward N., Basker S. (2009). GPS jamming and the impact on maritime navigation. J. Navig..

[8] Hambling D. (2011). GPS chaos: How a $30 box can jam your life. New Sci..

[9] Warburton J., Tedeschi C. GPS privacy jammers and RFI at Newark: Navigation team AJP-652 results. Proceedings of the 12th Int’l. GBAS Working Group Meeting (I-GWG-12).

[10] Pullen S., Gao G., Tedeschi C., Warburton J. The impact of uninformed RF interference on GBAS and potential mitigations. Proceedings of the 2012 International Technical Meeting of the Institute of Navigation.

[11] Seo J., Kim M. eLoran in Korea—Current status and future plans. Proceedings of the European Navigation Conference (ENC-GNSS).

[12] Drake S.P., Dogancay K. Geolocation by time difference of arrival using hyperbolic asymptotes. Proceedings of the 2004 IEEE International Conference on Acoustics, Speech, and Signal Processing.

[13] Ananthasubramanian B., Madlhow U. Cooperative localization using angle of arrival measurements in non-line-of-sight environments. Proceedings of the First ACM International Workshop on Mobile Entity Localization and Tracking in GPS-less Environments, Melt’08.

[14] Thompson R.J.R., Balaei A.T., Dempster A.G. Dilution of precision for GNSS interference localisation systems. Proceedings of the European Navigation Conf. (ENC GNSS2009), Parthenope Univ.

[15] Thompson R.J.R., Cetin E., Dempstre A.G. Detection and Jammer-to-Noise Ratio Estimation of Interferers Using the Automatic Gain Control. Proceedings of the International Global Navigation Satellite Systems Society IGNSS Symposium 2011, University of New South Wales.

[16] Bastide F., Akos D., Macabiau C., Roturier B. Automatic gain control (AGC) as an interference assessment tool. Proceedings of the 16th International Technical Meeting of the Satellite Division of The Institute of Navigation (ION GPS/GNSS 2003).

[17] Wang S., Inkol R. A near-optimal least squares solution to received signal strength difference based geolocation. Proceedings of the 2011 IEEE International Conference on Acoustics, Speech and Signal Processing (ICASSP).

[18] Thompson R.J.R., Cetin E., Dempstre A.G. Unknown Source Localization Using RSS in Open Areas in the Presence of Ground Reflections. Proceedings of the 2012 IEEE/ION Position, Location and Navigation Symposium.

[19] Thompson R.J.R. (2013). Detection and Localisation of Radio Frequency Interference to GNSS Reference Stations. Ph.D. Thesis.

[20] Bekcibasi U., Tenruh M. (2014). Increasing RSSI localisation accuracy with distance reference anchor in wireless sensor networks. Acta Polytech. Hung..

[21] Egenbråten S.A. (2015). RF Emitter Geolocation Using PDOA Algorithms and UAVs-A Strategy from Emitter Detection to Location Prediction. Master’s Thesis.

[22] Robertson A., Kompella S., Molnar J., Fu FDillon M., Perkins D. (2015). Distributed Transmitter Localization by Power Difference of Arrival (PDOA) on a Network of GNU Radio Sensors.

[23] Hu Y., Leus G. (2017). Robust Differential Received Signal Strength-Based Localization. IEEE Trans. Signal Process..

[24] Nyström M. (2017). GNSS Interference Localisation thought PDOA-Methods. Master’s Thesis.

[25] Zhou B., Chen Q., Xiao P. (2017). The Error Propagation Analysis of the Received Signal Strength-Based Simultaneous Localization and Tracking in Wireless Sensor Networks. IEEE Trans. Inf. Theory.

[26] Niu R., Vempaty A., Varshney P.K. (2018). Received-Signal-Strength-Based Localization in Wireless Sensor Networks. Proc. IEEE.

[27] Pu Y.C., You P.C. (2018). Indoor positioning system based on BLE location fingerprinting with classification approach. Appl. Math. Model..

[28] Li Y., He Z., Li Y., Gao Z., Chen R., El-Sheimy N. (2019). Enhanced Wireless Localization Based on Orientation-Compensation Model and Differential Received Signal Strength. IEEE Sens. J..

[29] Wu C., Wang X., Chenb M., Kim M.J. (2019). Differential received signal strength-based RFID positioning for construction equipment tracking. Adv. Eng. Inform..

[30] Xu S., Dogancay K. Optimal sensor development for 3D target localisation. Proceedings of the 2015 IEEE International Conference on Acoustics, Speech and Signal Processing (ICASSP).

[31] Xu S. (2020). Optimal Sensor Placement for Target Localization Using Hybrid RSS, AOA and TOA Measurements. IEEE Commun. Lett..

[32] Lee B.H., Park K.M., Kim Y.H., Kim S.C. (2021). Hybrid Approach for Indoor Localization Using Received Signal Strength of Dual-Band Wi-Fi. Sensors.

[33] Bo X., Razzaqi A.A., Wang X., Farid G. (2020). Optimal geometric configuration of sensors for received signal strength based cooperative localization of submerged AUVs. Ocean Eng..

[34] Alanezi M.A., Bouchekara H.R.E.H., Javaid M.S. (2021). Range-Based Localization of a Wireless Sensor Network for Internet of Things Using Received Signal Strength Indicator and the Most Valuable Player Algorithm. Technologies.

[35] Eshagh M. (2022). Optimisation of basepoints’ configuration in localisation of signal interference device. J. Surv. Eng..

[36] Eshagh M. (2022). An optimal design of GNSS interference localisation wireless security network based on time-difference of arrival for the Arlanda international airport. J. Geod. Sci..

[37] Ma F., Xu Y., Xu P. (2021). Research on the Minimum Size of Received Signal Strength Difference Localization Network. Int. J. Comput. Intell. Syst..

[38] Koch K.R. (2010). Parameter Estimation and Hypothesis Testing in Linear Models.

[39] Cooper M.A.R. (1987). Control Surveys in Civil Engineering.

[40] Xu P. (1989). Multi-Objective Optimal Second Order Design of Networks. Bull. Geod..

[41] Koch K.R. (1982). Optimization of the Configuration of Geodetic Networks. Dtsch. Geod. Komm..

[42] Koch K.R., Grafarend E.W., Sanso F. (1985). First Order Design: Optimization of the Configuration of a Network by Introducing Small Position Changes. Optimization and Design of Geodetic Networks.

[43] Kuang S. (1996). Geodetic Network Analysis and Optimal Design: Concepts and Applications.

